# Photodynamic Inactivation of *Legionella pneumophila* Biofilm Formation by Cationic Tetra- and Tripyridylporphyrins in Waters of Different Hardness

**DOI:** 10.3390/ijms22169095

**Published:** 2021-08-23

**Authors:** Martina Mušković, Iva Ćavar, Andrija Lesar, Martin Lončarić, Nela Malatesti, Ivana Gobin

**Affiliations:** 1 Department of Biotechnology, University of Rijeka, Radmile Matejčić 2, 51000 Rijeka, Croatia; martina.muskovic@biotech.uniri.hr (M.M.); iva.cavar@student.uniri.hr (I.Ć.); 2 Bioinstitut d.o.o., R. Steinera 7, 40000 Čakovec, Croatia; lesar@bioinstitut.hr; 3 Photonics and Quantum Optics Unit, Center of Excellence for Advanced Materials and Sensing Devices, Ruđer Bošković Institute, Bijenička Cesta 54, 10000 Zagreb, Croatia; martin.loncaric@irb.hr; 4 Department of Microbiology and Parasitology, Faculty of Medicine, University of Rijeka, Braće Branchetta 20, 51000 Rijeka, Croatia; ivana.gobin@medri.uniri.hr

**Keywords:** porphyrins, disinfection, *Legionella pneumophila*, biofilm, water hardness, singlet oxygen

## Abstract

The bacterium *Legionella pneumophila* is still one of the probable causes of waterborne diseases, causing serious respiratory illnesses. In the aquatic systems, *L. pneumophila* exists inside free-living amoebae or can form biofilms. Currently developed disinfection methods are not sufficient for complete eradication of *L. pneumophila* biofilms in water systems of interest. Photodynamic inactivation (PDI) is a method that results in an antimicrobial effect by using a combination of light and a photosensitizer (PS). In this work, the effect of PDI in waters of natural origin and of different hardness, as a treatment against *L. pneumophila* biofilm, was investigated. Three cationic tripyridylporphyrins, which were previously described as efficient agents against *L. pneumophila* alone, were used as PSs. We studied how differences in water hardness affect the PSs’ stability, the production of singlet oxygen, and the PDI activity on *L. pneumophila* adhesion and biofilm formation and in biofilm destruction. Amphiphilic porphyrin showed a stronger tendency for aggregation in hard and soft water, but its production of singlet oxygen was higher in comparison to tri- and tetracationic hydrophilic porphyrins that were stable in all water samples. All three studied porphyrins were shown to be effective as PDI agents against the adhesion of the *L. pneumophila* to polystyrene, against biofilm formation, and in the destruction of the formed biofilm, in their micromolar concentrations. However, a higher number of dissolved ions, i.e., water hardness, generally reduced somewhat the PDI activity of all the porphyrins at all tested biofilm growth stages.

## 1. Introduction

Photodynamic therapy (PDT) is used to treat various oncological and nononcological diseases by generating cytotoxic single oxygen (^1^O_2_) and other reactive oxygen species (ROS) at the site of light activation of a photosensitizer (PS) molecule [1]. Antibacterial photodynamic therapy (aPDT), also familiar under the name of photodynamic inactivation (PDI), can be used against various pathogens, so far mostly tested on *Escherichia coli* and *Staphylococcus aureus* [2,3], and for treating various infections, such as wound infections, acne, and periodontal diseases [4,5].

Some of the advantages of PDI over other antimicrobial therapies include the wide range of potential PSs that can be used, the limited toxicity of the treatment, and a low possibility to develop resistance to this therapy, due to its nonselective mode of action and reacting with lipids, proteins, and nucleic acids as part of the different biomolecular targets [5]. The rapidly growing field of application of PDI in disinfection and against biofilms includes the treatments of topical infections [6], oral biofilms in dentistry [7,8], and biofilms on prosthetic material/implants [9].

It is well known from the literature and research so far that all PSs, neutral, anionic, and cationic, can be effective against Gram-positive bacteria; however, only positively charged PSs seem to be effective alone against Gram-negative bacteria. This group of bacteria has two membranes separated by a peptidoglycan-containing periplasm, and so the outer membrane forms a barrier that can block many PSs from binding [10]. In the case of anionic or neutral PSs, outer membrane disrupting agents, such as ethylenediaminetetraacetic acid and calcium chloride, are necessary to enable their photodynamic action against Gram-negative bacteria [11]. It has been proposed that cationic porphyrins undergo a “self-promoted uptake pathway”, a mechanism that includes the electrostatic interaction with the outer membrane and results in its weakening, which increases the binding of the PS to the bacteria, enhancing the PDI activity without a pretreatment with “membrane softeners” [12].

*Legionella pneumophila* is a Gram-negative bacterium and a member of the class of opportunistic premise plumbing pathogens (OPPPs), a group of pathogens with common features that include the formation of biofilms and resistance to commonly used disinfection approaches such as the use of thermal treatment, chlorination, and biocides. Other OPPPs that pose a danger to human health include *Mycobacterium* spp. and *Pseudomonas aeruginosa* [13].

*Legionellae*, in water systems, mostly develop inside free-living amoebae or biofilms [14]. Biofilm, as an organized aggregate of microorganisms, can provide shelter from various antibiotics and a supply of nutrients for growth [15]. It was shown that *L. pneumophila* can adhere and form a biofilm on different materials, including plastic and stainless steel; however, some other materials such as copper may be used to inhibit adhesion [16]. Some physicochemical properties that can induce *L. pneumophila* biofilm formation are the stagnation of the water, the availability of the carbon that provides nutrients, and bivalent cations such as Mg^2+^ and Ca^2+^ that were shown to ease the attachment of the *L. pneumophila* to various materials [17].

Several treatments have been tested in the eradication of *L. pneumophila* biofilm, such as chlorination; use of phages; control of the carbon sources; or the use of the nanoparticles, different surfactants, antimicrobial peptides, or essential oils. However, none of these treatments induced a complete elimination, since the recolonization of the bacterium occurred in a short time after the end of the treatment [18].

In our previous work, three cationic pyridylporphyrins were investigated against *L. pneumophila* in the local tap water. All three porphyrins showed high PDI activity at low minimal effective concentration (MEC), but the highest activity, in concentration <0.1 μM, was shown with a tricationic porphyrin bearing a long alkyl chain, TMPyP3-C_17_H_35_ [19]. *L. pneumophila* strain 130b was used for all our previous studies; however, due to the increase in passages in laboratory conditions, phenotypic changes occurred, so for the biofilm studies, we used the clinical isolate of *L. pneumophila Philadelphia* strain, which is a common clinical isolate in Europe and the world. The difference between these two strains is not very well known, but it is possible that they differ in the lipopolysaccharide component of the outer membrane [20].

A higher amount of cations dissolved in water can positively affect the biofilm formation, due to salt precipitation and scale formation that facilitate *L. pneumophila* adhesion, and it also may improve colonization of biofilms by the bacteria [17]. In our previous work, we have observed small spectroscopic changes of tested porphyrins in tap water [19]; thus, we wanted to investigate in more detail the impact of water hardness on our porphyrins and their antibacterial activity. Herschmann et al., for example, investigated the physicochemical properties of tetracationic pyrid-4-ylporphyrin and its activity against *E. coli* in presence of various monovalent and bivalent cations [21]. They observed a high impact on singlet oxygen production and PDI only in presence of toxic metals; however, this motivated us to investigate the effect of ions naturally present in the raw groundwater in the treatment against *L. pneumophila* by PDI.

Therefore, the aim of our work that we present here was to investigate further the PDI potential of cationic PSs in *Legionella* control and their antibiofilm properties by investigating the PDI activity of the three above-mentioned cationic porphyrins in water samples of different hardness. As far as we know, this is the first study of the photodynamic effect on *Legionella* biofilm.

## 2. Results and Discussion

### 2.1. Physicochemical Properties of Water Samples

In our previous paper, we presented results that showed the high antibacterial efficacy of three cationic pyridylporphyrins and PDI against *L. pneumophila* in tap water and thus their significant potential for water treatments to control Legionella [19]. Therefore, we wanted to investigate further this potential, as well as the effects of PDI on *L. pneumophila* biofilm in water, using samples taken directly from the water wells. The idea was to investigate the effect of these compounds on the *Legionella* biofilm, with and without light activation of the PSs, in the raw groundwater and to examine the influence of the minerals present in different water samples in different amounts.

The main difference between the three different types of water used in this work was in the amount of calcium and magnesium ions dissolved in water, i.e., in the hardness of the water. Water hardness is usually described by the amount of dissolved calcium carbonate (CaCO_3_) in water, although other multivalent or divalent ions dissolved, such as, iron, barium, and zinc, can be taken into an account [22]. Water samples from water wells in Nedelišće and Prelog, two locations in Međimurska County (Croatia), were used as such without further manipulation; only a filter sterilization was performed before the use in experiments on *L. pneumophila* and adding the bacterium. The water sample from the water well in Prelog, with 403 mg/L CaCO_3_ dissolved, was throughout our studies considered as hard water (HW), while the water sample from the water well in Nedelišće, with 231 mg/L CaCO_3_ dissolved, was considered as soft water (SW). Demineralized water (DEMI) was used as a control, with a negligible amount of dissolved minerals (Table 1).

### 2.2. Spectroscopic Properties of the Tested PSs in Waters of Different Hardness

**Figure 1:**
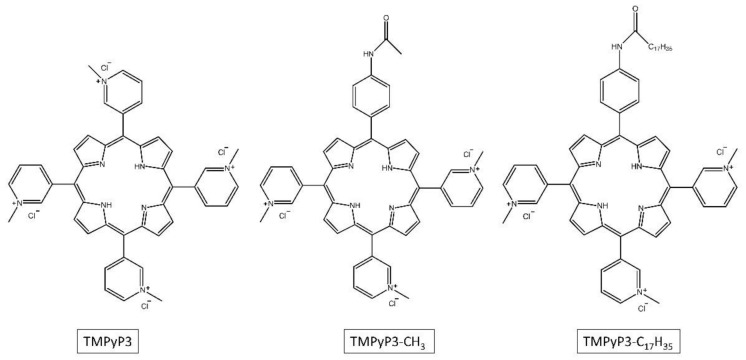
Structures of photosensitizers TMPyP3, TMPyP3-CH_3_, and TMPyP3-C_17_H_35_.

**Figure 2:**
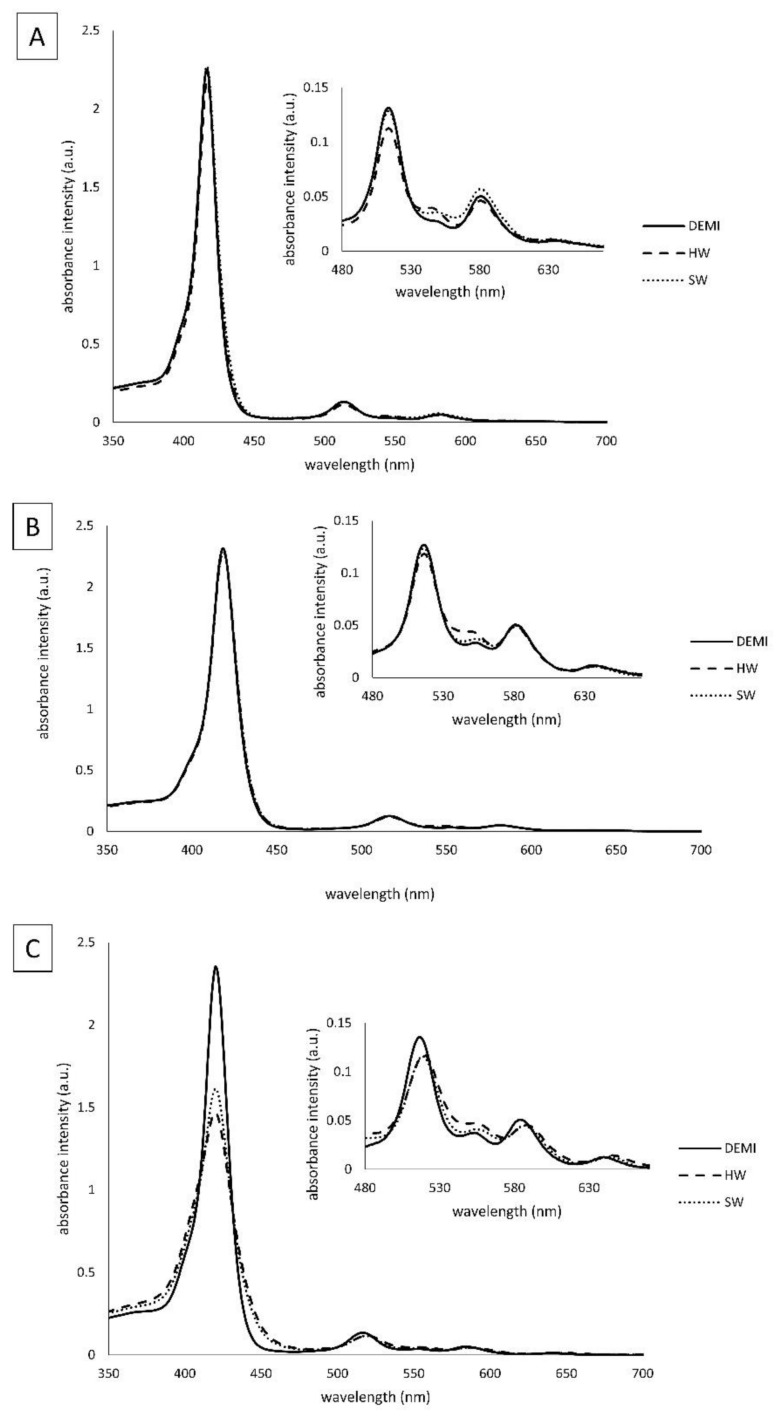
Absorption spectral characteristics of TMPyP3 (**A**), TMPyP3-CH_3_ (**B**), and TMPyP3-C_17_H_35_ (**C**) in DEMI (full line), soft water (dashed line), and hard water (dotted line). All measurements were performed in 10 μM solutions.

The spectroscopic properties of TMPyP3, TMPyP3-CH_3,_ and TMPyP3-C_17_H_35_ (Figure 1) were measured in water samples that contained different amounts of dissolved minerals (denoted as DEMI, SW, and HW). In the absorption spectra obtained in DEMI with all the compounds, there is a strong Soret band (with the molar absorption coefficients ε ~ 250 × 10^4^ M^−1^cm^−1^) at 416 nm for TMPyP3, at 418 nm for TMPyP3-CH_3_, and at 420 nm for TMPyP3-C_17_H_35_, and there are four Q bands from 500 to 650 nm. Q bands, which are normally seen as four defined sharp peaks in the spectra obtained in MeOH, here can be seen as a “stretched and broad” peak, which we observed also in our previous work where we tested these porphyrins in the tap water [19,23]. In the soft (SW) and hard (HW) water, small to negligible changes can be observed in the Q bands for compounds TMPyP3 and TMPyP3-CH_3_ (Figure 2A,B). On the other hand, the absorption spectra of TMPyP3-C_17_H_35_ obtained in DEMI, SW, and HW show more significant differences in both Soret band and Q bands (Figure 2C). The presence of minerals in water correlates with a decreasing intensity of the Soret band and its molar absorption coefficient (from 255 × 10^3^ to 159 × 10^3^ M^−1^cm^−1^) (Table S1), and a broadening of the Soret band occurs in SW and HW in comparison to DEMI. Besides a lower intensity, a small bathochromic shift can be seen for Q_y_(1-0) and Q_x_(1-0) electronic transitions. These changes in the absorption spectra may indicate the formation of nonspecific aggregates in the presence of dissolved minerals in water [24].

**Figure 3:**
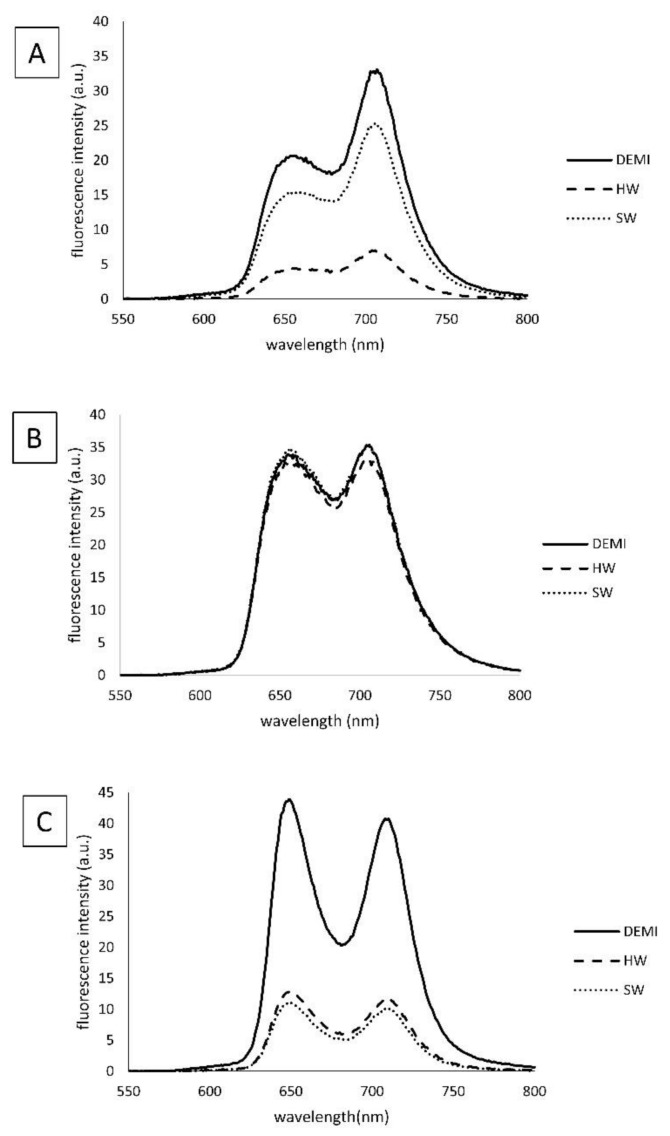
Fluorescence spectral characteristics of TMPyP3 (**A**), TMPyP3-CH_3_ (**B**), and TMPyP3-C_17_H_35_ (**C**) in DEMI (full line), soft water (dashed line), and hard water (dotted line). All measurements were performed in 1 μM solutions.

In the emission spectra, more significant differences can be seen in the fluorescence intensity for compounds TMPyP3-C_17_H_35_ and TMPyP3, while spectra for TMPyP3-CH_3_ show only small differences in different water samples at concentration 1 μM (Figure 3). Again, the intensity in SW and HW is decreased for TMPyP3-C_17_H_35_ in comparison to DEMI, which confirms the possibility of aggregation, presumably due to the presence of a long alkyl chain. The TMPyP3 spectra show a mild decrease in fluorescence intensity when collected in SW; however, they show a strong decrease and thus a very low intensity in HW (Figure 3A). Considering the structure of the molecule, its symmetry and π–π interactions may promote aggregation and consequently increase the fluorescence intensity, especially in the presence of ions such as in the SW and HW samples.

### 2.3. Photostability of the PSs in Different Water Samples

**Figure 4:**
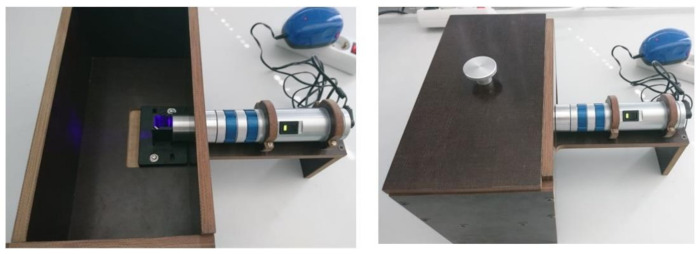
Illumination setup for the detection of singlet oxygen and photostability studies. The light source used is an LED-based source of violet light at 411 nm, with interchangeable fluence rates (3.5, 7, and 11 mW/cm^2^).

To investigate photostability, two 5-day-long experiments with repeated irradiation cycles (T1) and first-day-only irradiation cycles (T2) were carried out and compared to a nonirradiated sample that served as a control. A setup for irradiation, protected from external light, was used with an LED-based source of violet light (411 nm) and fluence rate 11 mW/cm^2^ (Figure 4).

**Figure 5:**
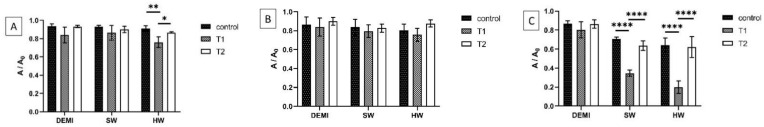
Photostability of TMPyP3 (**A**), TMPyP3-CH_3_ (**B**), and TMPyP3-C_17_H_35_ (**C**) measured in DEMI, SW, and HW. T1 represents a ratio of the absorbance recorded on the fifth and on the first day (A/A_0_) of 10 μM PS solution that was irradiated for 10 min every 24 h for 5 days (411 nm; total light dose 33 J/cm^2^), while T2 represents A/A_0_ after a 5-day-long experiment where each PS was irradiated for 10 min only the first day (411 nm; total light dose 6.6 J/cm^2^). Control represents PSs tested under the same conditions, protected from light. Data represent an average of triplicate measurements ± standard deviation (SD); **** *p* < 0.0001, ** 0.001 < *p* < 0.01, * 0.01 < *p* < 0.05.

The results of the photostability measurements after 5-day experiment are shown here as a ratio of the absorbance on the first and last (fifth) days of the experiment (A/A_0_) (Figure 5) and over time (Figures S1–S3). For TMPyP3 in DEMI and SW, there are no noticeable differences in absorption, and more than 85% of the initial absorbance (A_0_) remained in all the experiments (Figure 5A and Figure S1). A difference can be observed in the T1 experiment with the same PS in HW, where the sample was irradiated every day for 10 min. This decrease is significant in comparison to the dark control and the T2 experiment, which indicates that repeated irradiation together with HW can somewhat affect the stability of the molecule.

Most interesting, for TMPyP3-C_17_H_35_ only in DEMI, the water absorbance remained above 80% in both experiments and in the control (Figure 5C and Figure S3). The impact of repeated irradiation in comparison to one-time irradiation can be seen both in SW and HW, where the percentage of the initial absorbance in SW was 27% for T1 and 58% for T2, while in HW, it was 20% and 58% for T1 and T2, respectively. The impact of dissolved ions in water on the stability of this PS can be clearly seen in the increasing absorbance from DEMI to SW and mostly in HW. For example, control in DEMI stayed above 86%, while it was 70% in SW and HW; in T2, in DEMI, the water percentage of the absorbance intensity was again above 80%, while in both SW and HW, it was ~58% (Figure 5A).

For TMPyP3-CH_3_, no significant difference was observed, with all the absorbance intensities above 80% of A_0_ after 5 days of experiments in all water samples (Figure 5B and Figure S2), indicating the greatest photostability among all tested PSs, even in the presence of minerals. Considering the correlation of these results with the emission spectra shown in Figure 3, the decrease in (photo)stability of the tested compounds can be related to a higher tendency for aggregation.

### 2.4. Singlet Oxygen Production

To investigate the impact of water hardness on the production of singlet oxygen, the photodegradation of 9,10-anthracenediyl-bis(methylene)dimalonic acid (ABMDMA) was used. ABMDMA is known as a fluorescent dye that is soluble in water, but with a low reactivity, and it can capture only 2% of the produced singlet oxygen [25]. However, it is highly specific for singlet oxygen as opposed to other reactive oxygen species (ROS). In contrast, 1,3-diphenylisobenzofurane (DPBF), which is often used as a fluorescent probe in the measurements of the singlet oxygen production, reacts with the various ROS [26,27]. Here, we used the method modified from our previous work, where we used non-water-soluble 9,10-dimethylantracene (DMA) [28]. ABMDMA was used in a concentration of 6 μM, mixed with a 1 μM concentration of the PSs, and then irradiated for 10 min (411 nm, fluence rate 7 mW/cm^2^, light dose 4.2 J/cm^2^).

**Figure 6:**
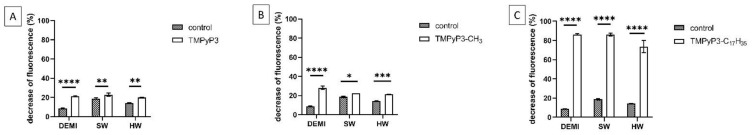
Singlet oxygen production of TMPyP3 (**A**), TMPyP3-CH_3_ (**B**), and TMPyP3-C_17_H_35_ (**C**) in different water samples (DEMI, SW, HW) (1 μM solution) measured by photodegradation of 9,10-anthracenediyl-bis(methylene)dimalonic acid (ABMDMA) (6 μM) after 10 min irradiation (411 nm, fluence rate 3.5 mW/cm^2^, total light dose 2.1 J/cm^2^). The photodegradation of ABMDMA without the presence of PSs in different water samples was used as a control. Data on graphs are presented as a measurement average, with error bars presenting SD; **** *p* < 0.0001, *** 0.0001 < *p* < 0.001, ** 0.001 < *p* < 0.01, * 0.01 < *p* < 0.05.

Interestingly, with TMPyP3-C_17_H_35_ (Figure 6C and Figure S6), a strong decrease in the ABMDMA fluorescence (>80% in all water samples) could be observed in comparison to TMPyP3 and TMPyP3-CH_3_, which showed small to medium effect (~20%) (Figure 6A,B). A higher production of singlet oxygen for those two PSs was observed only in their higher concentrations, above 3 μM (data not shown).

One of the possible reasons for the observed effect could be the amphiphilicity of TMPyP3-C_17_H_35_ due to the hydrophilic moiety based on tricationic pyridinium units and highly lipophilic moiety based on the long alkyl chain on the remaining aryl group in the porphyrin meso position. An unusual aggregation in water and formation of vesicles were previously described for an amphiphilic porphyrin with a similar structure [29]. Therefore, one possibility is that such aggregates may capture molecules of ABMDMA, consequently increasing the reaction of ABMDMA with singlet oxygen and the formation of nonfluorescent endoperoxide. Although it is known that aggregates usually decrease the singlet oxygen production in all solvents, a similar effect was shown by Rapozzi et al., who showed a dramatic decrease in pheophorbide A (PPa) singlet oxygen production in the phosphate buffer (ϕ < 0.01); however, the singlet oxygen production was high inside the liposomes and cells [30]. A higher production of ROS can have an impact on the stability of the molecule [31], so this could explain together the observed high production of singlet oxygen for TMPyP3-C_17_H_35_ and low photostability of the molecule, especially in cases of repeated irradiation of the solution.

The impact of the water hardness on the singlet oxygen production by PDT with TMPyP3 and TMPyP3-CH_3_ could not be noticed, due to the small production of singlet oxygen in tested PS concentration. A lower ^1^O_2_ production in HW, in comparison to SW and DEMI, can be observed for TMPyP3-C_17_H_35_, where the percentage of the fluorescence decrease was 73%, while it was around 86% in DEMI and SW. A higher light dose did not show a significant increase in singlet oxygen production for TMPyP3 or TMPyP3-CH_3_ (Figures S4 and S5).

According to the results of photostability and singlet oxygen production, as well as our previous results, it can be concluded that the amphiphilic TMPyP3-C_17_H_35_ is highly potent PS for PDI; however, it has a higher tendency for aggregation in the presence of various minerals dissolved in water, and its stability is strongly decreased, especially by repeated illumination with violet light. In contrast, both hydrophilic porphyrins (TMPyP3 and TMPyP3-CH_3_) showed higher stability for a long time, with and without light irradiation, and can possibly produce singlet oxygen in higher concentrations.

### 2.5. Minimal Effective Concentrations (MECs) and Photoinactivation (PDI) Studies of the PSs on Legionella pneumophila

**Figure 7:**
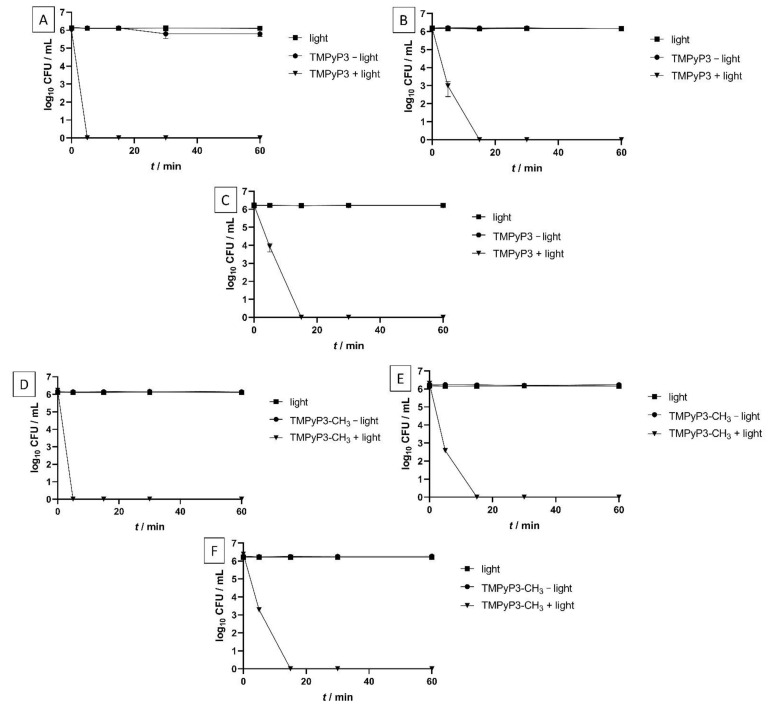

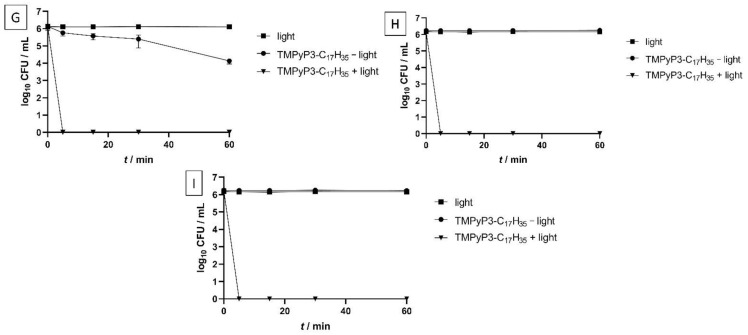
Photoinactivation studies of TMPyP3 (**A** (DEMI), **B** (SW), **C** (HW)), TMPyP3-CH_3_ (**D** (DEMI), **E** (SW), **F** (HW)), and TMPyP3-C_17_H_35_ (**G** (DEMI), **H** (SW), **I** (HW)) in 0.5 × MEC concentrations. The samples were irradiated for 60 min with violet light (395 nm, total light dose (after 60 min irradiation) 72 J/cm^2^). “PS−light” represents dark control, while “light” represents bacteria irradiated with light without presence of PS. All data are presented as an average  ± SD.

To determine the antibacterial activity of the PSs on *L. pneumophila* in different water samples, we determined the minimal effective concentrations (MECs), with and without irradiation, in these waters (Table 2). We also carried out (photo)inactivation studies using PSs against *L. pneumophila*, with and without irradiation (Figure 7).

From the obtained results, the MEC values increase for all PSs, under irradiation or without irradiation, with increasing water hardness. For tricationic PSs, the same MEC values were observed for both hydrophilic and amphiphilic porphyrins (0.78, 3.125, and 6.25 μM in DEMI, SW, and HW, respectively). The calculated MEC values for tetracationic PS (TMPyP3) were two or four times higher (3.125 μM in DEMI, 6.25 μM in SW, and 12.5 μM in HW) in comparison to the MEC values obtained for TMPyP3-CH_3_ and TMPyP3-C_17_H_35_. In our previous work, where *L. pneumophila* 130b strain was used, it was shown that in the tap water of Rijeka (considered as SW in this work) the calculated MEC for TMPyP3-C_17_H_35_ was in a nanomolar scale (0.024 μM) [19]. A somewhat dark toxicity in DEMI was also observed, with the MEC value of 3.125 μM for both TMPyP3-CH_3_ and TMPyP3-C_17_H_35_ and 6.25 μM for TMPyP3. Additionally, a low MEC concentration without irradiation was observed for TMPyP3-C_17_H_35_ in soft water (6.25 μM). This is in agreement with our previous work, where the strongest dark toxicity in tap water was also reported for amphiphilic TMPyP3-C_17_H_35_ (1.56 μM) [19].

PDI was studied using PS concentration 0.5 × MEC determined in SW under irradiation for each PS, which was 1.563 μM for TMPyP3-CH_3_ and TMPyP3-C_17_H_35_ and 3.125 μM for TMPyP3.

For porphyrins TMPyP3 and TMPyP3-CH_3_, it was shown that 100% of the bacteria were photoinactivated after 5 min of illumination with violet light (395 nm, 20 mW/cm^2^, light dose 6 J/cm^2^) in DEMI, and complete inactivation was achieved after 15 min in SW and HW (Figure 7). Except for TMPyP3-C_17_H_35_, there was no visible effect on bacteria in all water samples when a PS was used without photoactivation or when only light was applied, even after irradiation for 60 min (total light dose 72 J/cm^2^).

Amphiphilic PS, TMPyP3-C_17_H_35_, showed complete photoinactivation of *L. pneumophila* already after 5 min under irradiation (6 J/cm^2^) in all water samples. In a “light” control, where bacteria were irradiated with violet light without PS, no effect on bacteria was observed. However, in a “dark” control where *L. pneumophila* was treated with TMPyP3-C_17_H_35_ without irradiation, in DEMI, a 30% decrease in log_10_ CFU/mL was shown (Figure 7G). In SW and HW, “dark” toxicity of TMPyP3-C_17_H_35_ was not observed (Figure 7H,I).

### 2.6. Impact of PDI Activity on L. pneumophila Adhesion to Polystyrene

**Figure 8:**
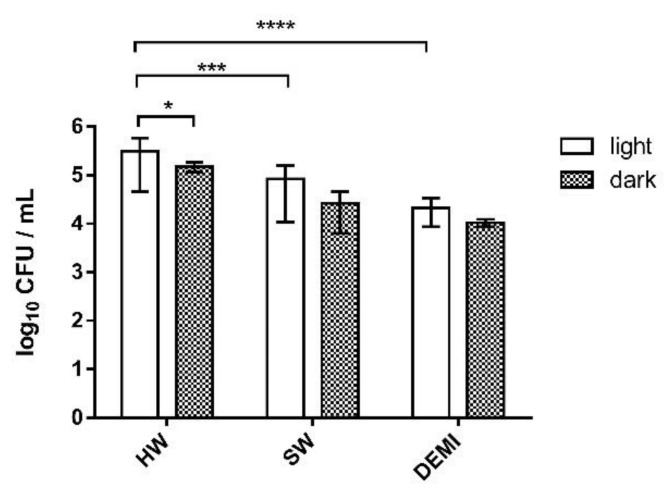
Adhesion of *L. pneumophila* to polystyrene in water samples of different hardness (HW, SW, and DEMI) after irradiation with violet light for 10 min (395 nm, total light dose 12 J/cm^2^) (=“light”) and without irradiation (=“dark”). All results are shown as an average of triplicate measurements and error bars presenting SD; **** *p* < 0.0001, *** 0.0001 < *p* < 0.001, * 0.01 < *p* < 0.05.

To investigate the effects of the PDI on *Legionella* biofilm using our porphyrin-type cationic PSs, we performed experiments of the adhesion of *L. pneumophila* on polystyrene, biofilm formation, and biofilm destruction. The first experiment included testing the impact of irradiation and water hardness on *Legionella* adhesion to polystyrene. We could observe that light has a small, almost negligible positive impact on the adhesion of *L. pneumophila*. Moreover, it could be seen that the presence and the number of dissolved ions can increase the number of colonies in the sample. The number of adherent bacteria, in an irradiated sample, was significantly higher in HW in comparison to the sample in SW and DEMI (Figure 8).

**Figure 9:**
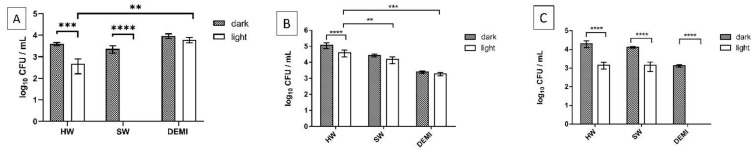
Impact of the PDI on adhesion of *L. pneumophila* to polystyrene in different water samples (DEMI, SW, HW) by using TMPyP3 (**A**), TMPyP3-CH_3_ (**B**) and TMPyP3-C_17_H_35_ (**C**) in concentration 0.25 × MEC determined in SW (0.782 μM for TMPyP3-CH_3_ and TMPyP3-C_17_H_35_, 1.563 μM for TMPyP3), after irradiation for 10 min with violet light (395 nm, total light dose 12 J/cm^2^). Treatment with PSs without irradiation was used as a “dark” control. All data are presented as mean ± SD; **** *p* < 0.0001, *** 0.0001 < *p* < 0.001, ** 0.001 < *p* < 0.01.

The impact of PDI on adhesion to polystyrene was tested in 0.25 × and 0.5 × MEC concentrations determined for each porphyrin in SW. TMPyP3-C_17_H_35_ in concentration 0.25 × MEC showed 100% inactivation of the adhesion in DEMI, while TMPyP3-CH_3_ showed ~30% decrease in comparison to control (Figure 9B,C). Negligible difference in DEMI was observed for TMPyP3 (Figure 9A). Additionally, somewhat “dark toxicity” was detected in DEMI for porphyrins TMPyP3-CH_3_ and TMPyP3-C_17_H_35_, with a decrease of 1 log_10_ CFU/mL in concentration 0.25 × MEC (Figure 9B,C). In SW and HW, a similar PDI effect was shown in both water samples for TMPyP3-C_17_H_35_, where the decrease was ~30%. A negligible decrease in comparison to the “light” control was observed for porphyrin TMPyP3-CH_3_ in SW and HW. In SW, porphyrin TMPyP3 showed complete inactivation of the adhesion, while in HW, ~50% decrease was observed (Figure 9A).

In a concentration of 0.5 × MEC, a complete inactivation of adhesion was shown for all tested porphyrins in DEMI and SW, while in HW, a ~50% decrease was observed for porphyrin TMPyP3-CH_3_ and a 100% inactivation was observed for PSs TMPyP3-C_17_H_35_ and TMPyP3. A complete inactivation without light irradiation, “dark toxicity”, was observed for all porphyrins in DEMI (Figure S7).

### 2.7. Impact of PDI Activity on L. pneumophila Biofilm Formation

**Figure 10:**
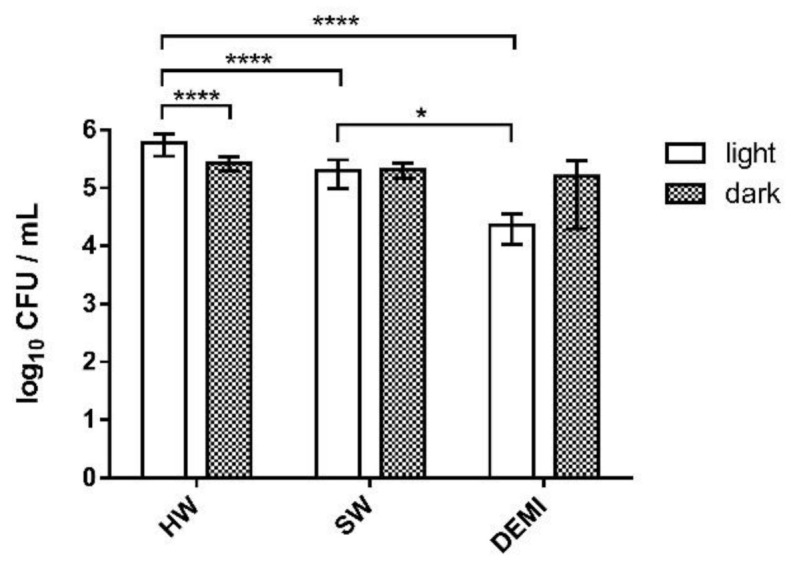
*L. pneumophila* biofilm formation in water samples of different hardness (HW, SW, and DEMI) with (=“light”) and without irradiation (=“dark”). For the samples that were illuminated, an LED-based source of violet light was used for 10 min treatment (395 nm, total light dose 12 J/cm^2^). All data are presented as an average ± SD; **** *p* < 0.0001, * 0.01 < *p* < 0.05.

The impact of PDI activity was also investigated on early-stage biofilm, i.e., on biofilm formation. Firstly, we tested the impact of light and dissolved minerals in water on biofilm formation, and no difference could be observed between irradiated and nonirradiated samples and between water samples of different hardness. The only difference could be seen on the sample irradiated in DEMI, where a 1-log decrease was detected (Figure 10).

**Figure 11:**
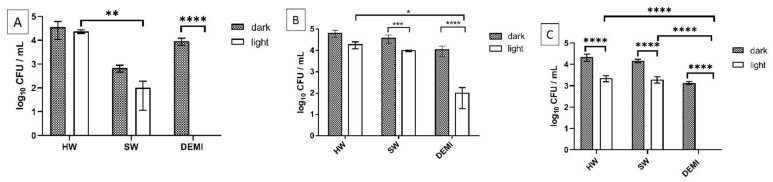
Impact of the PDI on *L. pneumophila* biofilm formation in different water samples (DEMI, SW, HW) by using TMPyP3 (**A**), TMPyP3-CH_3_ (**B**), and TMPyP3-C_17_H_35_ (**C**) in concentration 0.25 × MEC determined in SW (0.782 μM for TMPyP3-CH_3_ and TMPyP3-C_17_H_35_, 1.563 μM for TMPyP3), after irradiation for 10 min with violet light (395 nm, total light dose 12 J/cm^2^). The treatment with PSs without irradiation was used as a “dark” control. All data are presented as mean ± SD; **** *p* < 0.0001, *** 0.0001 < *p* < 0.001, ** 0.001 < *p* < 0.01, * 0.01 < *p* < 0.05.

The tested PSs showed also a high PDI efficiency on *L. pneumophila* biofilm formation. PDI with tetracationic TMPyP3 resulted in a complete eradication of *L. pneumophila* early-stage biofilm in DEMI, already in concentration 1/4 × MEC. However, in SW ~ 60% was inactivated, while in HW a negligible inactivation (<1-log decrease) was observed (Figure 11A). In the concentration of 1/2 × MEC, the activity of TMPyP3 in HW increased, which led to a decrease in log_10_ CFU for >50% (Figure S8A). A small dark toxicity was observed for TMPyP3 in concentration 1/4 × MEC, especially in SW with a >2-log decrease in comparison to biofilm that was kept in the dark or biofilm that was irradiated with violet light for 10 min (Figure 10, SW). In DEMI and HW, the observed dark toxicity was lower (~1-log decrease) in comparison to SW (Figure 11A). With TMPyP3 in a concentration of 1/2 × MEC, a strong dark toxicity was observed in both SW and DEMI, decreasing the number of bacteria by >50% (Figure S8A).

A complete inactivation occurred in DEMI after the treatment with TMPyP3-C_17_H_35_ (Figure 11C). In HW and SW, a similar effect with a 40% decrease after the treatment with 1/4 × MEC was observed. Interestingly, after increasing the concentration of the TMPyP3-C_17_H_35_ to 1/2 × MEC, a complete deactivation of the biofilm formation occurred in SW, while a negligible impact was shown in HW (Figure S8C). Additionally, a complete inactivation without illumination, i.e., dark toxicity, was observed after the treatment with 1/2 × MEC of TMPyP3-C_17_H_35_ in DEMI (Figure S8C).

TMPyP3-CH_3_ in 1/4 × MEC concentration showed a 3-log decrease in DEMI, while in SW and HW, the PDI did not result in such a strong effect against biofilm formation (Figure 11B). In concentration 1/2 × MEC, a complete inactivation of the biofilm formation occurred in DEMI and SW water, while in HW, a 1-log decrease was shown after irradiation. A strong dark toxicity was observed in DEMI (Figure S8B).

### 2.8. L. pneumophila Biofilm Destruction in Different Water Hardness

**Figure 12:**
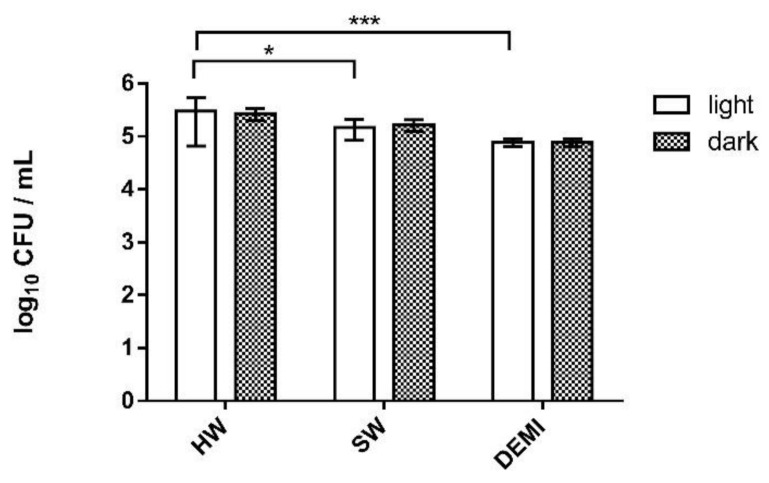
Impact of water hardness (HW, SW, and DEMI) and violet light irradiation on *L. pneumophila* biofilm. For the light treatments, irradiation with violet light was used for 10 min (395 nm, total light dose 12 J/ cm^2^). Results are presented as an average with SD on error bars; *** 0.0001 < *p* < 0.001, * 0.01 < *p* < 0.05.

As in the experiments of the adhesion and biofilm formation, we also tested the impact of light and water hardness on formed *L. pneumophila* biofilm. We could observe that water hardness has a small impact on *L. pneumophila* biofilm growth, the difference being lower than 0.5 log. Additionally, 10 min light irradiation did not show an impact on *L. pneumophila* biofilm growth (Figure 12).

**Figure 13:**
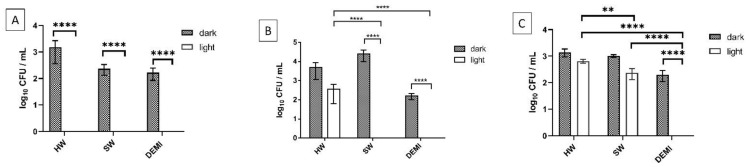
Destruction of the *L. pneumophila* biofilm with PDT using TMPyP3 (**A**), TMPyP3-CH_3_ (**B**), and TMPyP3-C_17_H_35_ (**C**) in concentration 1 × MEC determined in SW (3.125 μM for TMPyP3-CH_3_ and TMPyP3-C_17_H_35_, 6.250 μM for TMPyP3). For the light treatments, irradiation with violet light was used for 10 min (395 nm, total light dose 12 J/ cm^2^). “Dark” control represents the treatments with PSs, without irradiation. Results are presented as an average with SD on error bars; **** *p* < 0.0001, ** 0.001 < *p* < 0.01.

PDI activity was tested in concentrations 1 × MEC and 2 × MEC of PSs against *L. pneumophila* biofilm. A strong impact was observed already at 1 × MEC concentrations for all PSs in DEMI where 100% of the biofilm was destroyed (Figure 13). No biofilm could be detected after the treatment with light and TMPyP3 in 1 × MEC concentration in all water samples (HW, SW, and DEMI) (Figure 13A). Interestingly, TMPyP3-CH_3_ showed stronger activity on *L. pneumophila* biofilm in comparison to the amphiphilic analog by showing a complete inactivation in SW and a >50% decrease in HW (Figure 13B). In contrast, TMPyP3-C_17_H_35_ showed a 2.5-log decrease after the treatment in SW and a 2-log decrease in HW (Figure 13C).

An improved activity in 2 × MEC concentration was only shown after the treatment with TMPyP3-C_17_H_35_ in SW where complete destruction of the biofilm occurred. Moreover, dark toxicity with complete destruction of the biofilm for all porphyrins in 2 × MEC concentration in DEMI was observed (Figure S9).

It is generally accepted that the number of positive charges can increase the activity against Gram-negative bacteria; however, we must point out here that the 2× higher concentration of symmetrical, tetracationic TMPyP3 in comparison to both asymmetric, tricationic porphyrins may play a role in the observed higher antibacterial activity in all the treatments on biofilm adhesion, biofilm formation, and the destruction of biofilm. As in our previous studies, between the two tricationic porphyrins, amphiphilic TMPyP3-C_17_H_35_ with a long alkyl chain proved to be more active than hydrophilic TMPyP3-CH_3_, except in the destruction of the formed biofilm in the presence of minerals, which may be related to the demonstrated reduced photostability of TMPyP3-C_17_H_35_ in SW and HW.

Our results showed that water hardness does not strongly affect *Legionella* biofilm or biofilm formation; however, a small increase proportional to the amount of dissolved minerals in water can be observed both in the samples irradiated with violet light and the samples kept in the dark. As we mentioned at the beginning, calcium, magnesium, and organic carbon can increase the number of *Legionella* in the water and increase the adherence to various surfaces [17]. Bargellini and collaborators investigated the impact of various metal traces, hot water, and heterotrophic plate counts on *Legionella* contamination and growth. In accordance with our results, they concluded that water hardness does not have an impact on *Legionella* growth; however, water hardness can increase *Legionella* occurrence in water samples [32].

Various PSs have been tested as PDI agents in the destruction of biofilms. As an example, a phenothiazinic PS, methylene blue O (MBO), in ethanol formulation showed high *Pseudomonas aeruginosa* biofilm destruction [33]. Another example is erythrosine-mediated PDI against *Acetobacter baumannii*, a bacterium that can cause various skin infections and ventilator-associated pneumonia. It was shown that erythrosine alone did not affect the *A. baumannii* biofilm; however, in a combination with acetic acid and chitosan, it was considered a promising therapy [34]. Additionally, Rose Bengal (RB) and a fullerene were tested against two isolates of *Enterococcus faecium* and *E. faecalis*, Gram-positive bacteria that can cause various infections [35].

Porphyrin PSs have been widely tested in the treatment of *Pseudomonas aeruginosa*, another Gram-negative OPPP. Orlandi et al. recently tested the effects of a group of 13 diaryl porphyrins on biofilm formation and eradication of biofilm mass [36]. It was shown that diaryl porphyrins bearing two positively charged pyridyl groups on the opposite side of the porphyrin core have the highest antibiofilm properties and can inhibit the biofilm formation and have a mild effect on the formed biofilm [36]. The authors presumed that the highest antibiofilm properties are due to the positive charge of the pyridyl groups being delocalized and directly coordinated with the tetrapyrrole system of porphyrin. Another work, by Patel et al., confirms the importance of cationic groups in the molecular structure in eradicating *P. aeruginosa* biofilms by using 5,10,15-tris (*N*-methyl pyridyl)-20-pentafluoro phenyl porphyrinatozincTris-4-methylbenzenesulfonate (ZnPor) for PDI. ZnPor in concentrations 16 μg/mL showed a high impact on the eradication of the 10–16 h old biofilm and on the detachment of the matrix [37].

## 3. Materials and Methods

### 3.1. Porphyrins Used as PSs

The porphyrins used in this work are tetracationic TMPyP3 and two tricationic porphyrins, TMPyP3-CH_3_ and TMPyP3-C_17_H_35_, that were previously synthesized by our group [19].

### 3.2. Water Samples

The water samples used in this work were taken in Croatia, Međimurska County, from water wells in Prelog and Nedelišće. The water samples were taken before the treatment of water conditioning, and no additional treatments were done prior to the use. The physicochemical properties of the water samples were determined by the laboratory Međimurske Vode d.o.o. (in Čakovec, Croatia) and using portable HQ30D Flexi meter with changing probes for measuring conductivity, pH, and dissolved oxygen concentration (Hach, Loveland, CO, USA). In this work, we used the terms soft water (SW) and hard water (HW) for water samples from water wells Prelog and Nedelišće, respectively, based on the ion concentration and water conductivity. In addition to water samples from the wells, demineralized water (DEMI) was also used (Table 1).

Water samples without further purification were used in the experiments to determine the spectroscopic properties, the photostability of the compounds, and the amount of singlet oxygen produced by PDT. For in vitro biological experiments, all water samples were sterilized by filtration through FilterBio Sterile Syringe Filter (Budapest, Hungary, 0.45 µm, 25 mm) prior to their use. Sterile tap water (autoclaved at 121 °C for 15 min, cooled to at 25 °C, and stored at 4 °C), was used for rinsing and preparing 10-fold serial dilutions for determining the number of cultivable bacteria.

### 3.3. Light Sources

The LED-based sources of violet light used in this work were constructed and calibrated at the Center of Excellence for Advanced Materials and Sensors at the Ruđer Bošković Institute, Zagreb, Croatia. For singlet oxygen detection and photostability measurements, a light source at 411 nm with fluence rates of 3.5, 7, and 11 mW/cm^2^ was used. A light source for irradiation at 395 nm and fluence rate of 20 mW/cm^2^ was used in all biological in vitro experiments testing PDI against *L. pneumophila* and biofilm formation.

### 3.4. Spectroscopic Properties

For spectroscopic analysis, stock solutions of the PSs were prepared in DEMI (~100 μM) and diluted in DEMI, SW, or HW to final, tested concentrations. UV-Vis measurements were performed on Cary 60 spectrophotometer, and for fluorescence measurements, Cary Eclipse was used (Agilent Technologies, Santa Clara, CA, USA). The wavelength span for UV-Vis was from 350 to 500 nm and for fluorescence measurements was from 550 to 800 nm. The Soret band wavelength of each PS was used as the excitation wavelength for fluorescence measurements.

### 3.5. Photostability Measurements

The photostability of the PSs was measured as a decrease in the Soret band intensity over 5 days. Stock solutions of porphyrins were prepared in DEMI (~2 mM) and diluted in HW, SW, or DEMI to the final concentration. The first test (T1) consisted of illuminating the porphyrin solution (10 μM) with violet light for 10 min every 24 h for 5 days (411 nm; fluence rate: 11 mW/cm^2^; total light dose: 33 J/cm^2^), while in the second test (T2), porphyrin solutions (also 10 μM) were illuminated only on the first day for 10 min (411 nm; fluence rate: 11 mW/cm^2^, total light dose: 6.6 J/cm^2^). Detection time points were at 0, 5, and 10 min of irradiation. Between the illumination sections and the absorbance measurements, samples were kept in dark at room temperature. Dark control was obtained for porphyrin solutions in all 3 types of water by keeping the solutions in dark at room temperature all the time and measuring a decrease in the Soret band intensity every 24 h for 5 days.

### 3.6. Singlet Oxygen (^1^O_2_) Detection

Singlet oxygen production was evaluated by measuring the relative photodegradation of 9,10-anthracenediyl-bis(methylene)dimalonic acid (ABMDMA) (Alfa Aesar, Ward Hill, MA, USA), a commercially available fluorescent probe. In the presence of singlet oxygen, ABMDMA reacts via [4 + 2] Diels–Adler reaction and forms a nonfluorescent endoperoxide product. Relative fluorescence decrease was used for being proportional to the formation of the endoperoxide product, as corresponding to the amount of produced singlet oxygen.

Stock solutions of porphyrins and ABMDMA were prepared in DMSO (1 mM) (Sigma Aldrich, St. Louis, MO, USA), due to lack of solubility of ABMDMA in DEMI, and then dissolved in different water samples to final concentrations. Porphyrins in 1 μM concentration and 5 μM ABMDMA solution were mixed in ratio 1:1 and transferred to a 1 cm quartz cuvette (Hellma Analytics, Müllheim, Germany). The solution was irradiated for 10 min (411 nm; fluence rates 3.5, 7, and 11 mW/cm^2^ with final light doses of 2.1, 4.2, and 6.6 J/cm^2^, respectively) at room temperature under constant stirring. Fluorescence decrease was measured at 431 nm every 60 s. ABMDMA in different water samples without the PSs was used as a control. All measurements were performed in duplicate.

Results were presented as a percentage (%) of fluorescence decrease by calculating area under the curve (*AUC*) of measured decay according to the following formula:

(1) AUC= AA0 s+AA0f2×t2−t1

*AUC*—area under the curve;

AA0 s—ratio of the initial absorbance at beginning of the 60 s interval;

AA0 f—ratio of the initial absorbance at the end of the 60 s interval;

*t*2—time at the end of the interval;

*t*1—time at the beginning of the interval.

### 3.7. Bacteria Strain and Growth Conditions

Throughout the conduction of the PDI experiments, the clinical isolate of *Legionella pneumophila* serogroup 1, strain *Philadelphia* ST1, was used. The clinical isolate of *L. pneumophila* was obtained courtesy of Prof. Darja Keše from the University of Ljubljana, Slovenia. The bacteria were stored in 10% glycerol broth at −80 °C and routinely cultured on buffered yeast extract agar (BCYE) (Oxoid, Altrincham, UK) for 3–5 days at 35 ± 2 °C. Once cultured on BCYE, the bacteria were resuspended in sterile deionized water. The optical density at 600 nm (OD_600_) was measured for the prepared bacterial suspension to adjust the concentration of the stock suspension to approximately 1 × 10^9^ CFU/mL (OD_600_ measured 1 corresponded to 1 × 10^9^ CFU/mL). The concentrations of working suspensions (1.0 × 10^6^ CFU/mL) in water samples of different hardness were adjusted by preparing 10-fold serial dilutions. The results of the PDI studies were acquired by determining the number of cultivable bacteria in 10-fold serial dilutions on BCYE agar.

### 3.8. Determining Minimum Effective Concentration (MEC) of the PSs in Water Samples of Different Hardness

The MEC values for TMPyP3-C_17_H_35_, TMPyP3-CH_3_, and TMPyP3 were obtained using a microdilution technique in deionized water, soft water (SW), and hard water (HW). Methods used to determine MEC and PDI activity were previously described [19]. Two-fold serial dilutions (50 to 0.049 µM) of the PSs were prepared in sterile 96-well microtiter plates (Syntesis Padova Italy) for each water sample and mixed with equal volumes of 1.0 × 10^6^ CFU/mL bacterial suspensions per well. Afterward, bacterial suspensions and 2-fold serial dilutions of the PSs were incubated and mixed at room temperature for 30 min without light. After incubation in the dark, bacteria in 2-fold serial dilutions were exposed to the light source for 10 min (395 nm, total light dose 12 J/cm^2^). After irradiation, the samples were incubated for 24 h in the dark at 35 ± 2 °C, inoculated onto BCYE agar the following day, and incubated for another 3–5 days at 35 ± 2 °C. The MEC values were the lowest concentrations of the PSs in each water sample that yielded negative subcultures on BCYE agar. The toxicity of the PSs to bacterial cells in the dark for each water sample was determined following the same procedure, excluding exposure to the light source.

### 3.9. PDI Studies in Water Samples of Different Hardness

The bacterial suspensions (10^6^ CFU/mL) prepared in different water samples were mixed with the PSs also prepared in different water samples (0.5 × MEC determined for SW: 1.563 µM for TMPyP3-CH_3_ and TMPyP3-C_17_H_35_ and 3.125 µM for TMPyP3). After incubation and mixing at room temperature for 30 min in the dark, the samples were illuminated with violet light (395 nm) for different periods of time (0, 5, 15, 30, and 60 min, corresponding to a total light dose of 0, 6, 18, 36, and 72 J/cm^2^, respectively). Following each of the illumination periods, 10-fold serial dilutions of the samples were prepared and inoculated onto BCYE agar and incubated for 3–5 days at 35 ± 2 °C. After incubation, the number of cultivable bacteria was determined, and the obtained results were presented as survival curves of the bacterium (CFU/mL) dependent on the total dose of light (0–72 J/cm^2^).

### 3.10. Antiadhesion and Antibiofilm Properties of the PSs in Water Samples of Different Hardness on Polystyrene

Equal volumes of the bacterial suspensions (10^6^ CFU/mL) and solutions of the PSs were prepared in the water samples of different hardness and then mixed in sterile 96-well microtiter plates (Syntesis, Padova, Italy). The final concentrations of the PSs, once they were mixed with bacterial suspensions, were 0.5 × MEC (1.563 µM for TMPyP3-CH_3_ and TMPyP3-C_17_H_35_ and 3.125 µM for TMPyP3) and 0.25 × MEC (0.782 µM for TMPyP3-CH_3_ and TMPyP3-C_17_H_35_ and 1.563 µM for TMPyP3) determined for SW. The samples were incubated, while stirring, at room temperature for 30 min without light. After incubation, the samples were irradiated for 10 min (395 nm; total dose of light 12 J/cm^2^) and incubated for 24 h at 35 ± 2 °C. The following day, samples were washed 2 times with sterile tap water, and microtiter plates with samples were placed in the ultrasound bath (Bandelin-BactoSonic, Berlin, Germany) for 1 min (*p* = 200 W, *f* = 40 kHz). After 10-fold serial dilutions of the samples were prepared, the dilutions were inoculated onto BCYE agar and incubated for 3–5 days at 35 ± 2 °C. The number of cultivable bacteria was determined after incubation.

A similar procedure was conducted to test the antibiofilm properties of the PSs. The bacterial suspensions (10^6^ CFU/mL) and the porphyrin solutions (final concentration 0.5 × MEC and 0.25 × MEC determined for soft water) were mixed, incubated with stirring at room temperature without light, and irradiated for 10 min. After irradiation, the samples were incubated for 5 days at 35 ± 2 °C. Following the incubation, samples were washed 2 times with sterile tap water and treated in an ultrasound bath; afterward, 10-fold serial dilutions were prepared. The dilutions were then inoculated onto BCYE agar to determine the number of cultivable bacteria.

### 3.11. Effectiveness of the PSs and Their PDI Activity on Biofilm Destruction in Waters of Different Hardness

The bacterial suspensions (10^6^ CFU/mL) were placed in sterile 96-well microtiter plates (Synthesis, Italy) and incubated for 5 days at 35 ± 2 °C. After 5 days, biofilm in microtiter plates was washed 2 times with sterile tap water, and solutions of the PSs (1 × MEC (3.125 µM for TMPyP3-CH_3,_ and TMPyP3-C_17_H_35_ and 6.250 µM for TMPyP3) and 2 × MEC (6.250 µM for TMPyP3-CH_3_, and TMPyP3 and 12.500 µM for TMPyP3-C_17_H_35_) determined for SW) were added in wells where biofilm had formed. The bacterial suspensions and the porphyrin solutions were prepared in the water samples of different water hardness. The samples were incubated with stirring for 30 min at room temperature without light and exposed to the light source for 10 min (395 nm, total dose of light 12 J/cm^2^). After irradiation, the samples were incubated for 24 h at 35 ± 2 °C, washed 3 times with sterile tap water, and treated in the ultrasound bath for 1 min (*p* = 200 W, *f* = 40 kHz); then, 10-fold serial dilutions were prepared, inoculated onto BCYE agar, and incubated for 3–5 days at 35 ± 2 °C. The number of cultivable bacteria was determined following incubation.

### 3.12. Statistics

The statistical analysis in this work was performed using the program GraphPad Prism 8. All data were presented as average of repeated measurements with standard deviation on error bars. In all experiments, results were analyzed using two-way analysis of variance (ANOVA), where differences were marked as statistically significant if α < 0.05. Level of significance was presented in four levels: **** (*p* < 0.0001), *** (0.0001 < *p* < 0.001), ** (0.001 < *p* < 0.01), * (0.01 < *p* < 0.05).

## 4. Conclusions

In conclusion, PDI can be used as a treatment for *L. pneumophila* in waters of different hardness. The highest impact, as in our previous work, was shown with TMPyP3-C_17_H_35_, a tricationic porphyrin with a long alkyl chain. This PS showed the highest singlet oxygen production and PDI activity in all experiments including *L. pneumophila* biofilm. However, its stability strongly decreases after repeated irradiation cycles and in hard water.

On the other side, tetracationic TMPyP3 also showed a strong impact on *L. pneumophila* adhesion and biofilm formation and in the destruction of the formed biofilm; however, its MEC value obtained in SW was 2 times higher in comparison to both tricationic porphyrins. Finally, water hardness decreased the efficiency of all porphyrins against *L. pneumophila* and its biofilm since the highest PDI activity for each of them was observed in DEMI.

The results obtained suggest that the hydrophilic PSs could be used where the PDI action against *Legionella* biofilm over a long period of time and repetition of illumination is required, while the amphiphilic PS could be used where high efficiency in a short time is required. Since the work presented here is an initial study on the use of PDI to control *Legionella* biofilm formation in water, further research is needed to investigate these possibilities.

## Figures and Tables

**Table 1:** General properties of the water samples used in this work. Soft water (SW) and hard water (HW) were taken from water wells in Nedelišće and Prelog in Međimurska County (Croatia).

Title 1	Water Samples		
	DEMI	SW	HW
CaCO_3_ (mg/L) ^a^	/	231	403
pH ^a,b^	7.00	7.66	7.43
Conductivity (S/cm) (t = 20 °C) ^b^	4	403	678
Cl^−^ (mg/L) ^a^	/	8.53	20.08
K^+^ (mg/L) ^a^	/	1.7	1.8
Na^+^ (mg/L) ^a^	/	5.1	5.2
Mg^2+^ (mg/L) ^a^	/	7.7	23.3
Ca^2+^ (mg/L) ^a^	/	/	112.0
Hardness (mg/L) ^a^	0.2	240	396
Oxygen levels (mg/L) ^b^	2.55	9.54	9.80

^a^ Parameters measured in the laboratory of Međimurske vode d.o.o. ^b^ Parameters measured using HQ30D Flexi meter with changing probes.

**Table 2:** Minimal effective concentration (MEC) of TMPyP3, TMPyP3-CH_3_, and TMPyP3-C_17_H_35_ determined in DEMI after irradiation for 10 min with violet light (395 nm, 12 J/cm^2^).

		MEC (μM)	
		Dark	*λ* = 395 nm
TMPyP3	DEMI	6.250	3.125
	SW	>5	6.250
	HW	>25	12.50
TMPyP3-CH_3_	DEMI	3.125	0.780
	SW	>5	3.125
	HW	>25	6.250
TMPyP3-C_17_H_35_	DEMI	3.125	0.780
	SW	6.250	3.125
	HW	>25	6.250

## Data Availability

The data that support the findings of this study are available from the corresponding author upon reasonable request.

## Supplementary Materials

The following are available online at https://www.mdpi.com/article/10.3390/ijms22169095/s1.
