# Supplementary material for: Photodynamic Inactivation of Legionella pneumophila Biofilm Formation by Cationic Tetra- and Tripyridylporphyrins in Waters of Different Hardness
Source: Int J Mol Sci. 2021 Aug 23;22(16):9095. doi: 10.3390/ijms22169095 (PMC8396483; doi:10.3390/ijms22169095)
Supplement: Supplementary file 1 [file ijms-22-09095-s001.zip › ijms-1343002-supplementary.pdf]

Article

Photodynamic Inactivation of *Legionella Pneumophila* Biofilm Formation by Cationic Tetra- and Tripyridylporphyrins in Waters of Different Hardness

Martina Mušković [1], Iva Ćavar [1], Andrija Lesar [2], Martin Lončarić [3], Nela Malatesti [1,*] and Ivana Gobin [4]

[1] Department of Biotechnology, University of Rijeka, Radmile Matejčić 2, Rijeka 51000, Croatia; martina.muskovic@biotech.uniri.hr (M.M.); iva.cavar@student.uniri.hr (I.Ć.)
[2] Bioinstitut d.o.o., R. Steinera 7, Čakovec 40000, Croatia; lesar@bioinstitut.hr
[3] Photonics and Quantum Optics Unit, Center of Excellence for Advanced Materials and Sensing Devices, Ruđer Bošković Institute, Bijenička cesta 54, Zagreb 10000, Croatia; martin.lončarić@irb.hr
[4] Department of Microbiology and Parasitology, Faculty of Medicine, University of Rijeka, Braće Branchetta 20, Rijeka 51000, Croatia; ivana.gobin@medri.uniri.hr
* Correspondence: nela.malatesti@biotech.uniri.hr; Tel.: +385-51-584-585; Fax: +385-51-584-599

Table S1. Absorption and fluorescence properties of TMPyP3 (A), TMPyP3-CH_3 (B) and TMPyP3-$C_{17}H_{35}$ (C) in waters of different hardness (DEMI, HW and SW). Fluorescence was measured at concentration 1 μM after excitation at Soret band wavelength.

A	Absorbance properties λ_{max} (nm) (ε x 10^3 $M^{-1}cm^{-1}$)					Fluorescence propeties	
	Soret	Q_y (1-0)	Q_y (0-0)	Q_x(1-0)	Q_y(0-0)	λ_{max} (nm)	Stokes shift
**DEMI**	416 (247.2)	514 (14.4)	550 (2.3)	580 (3.0)	650 (0.7)	656, 708	6
**HW**	417 (237.7)	514 (12.6)	544 (3.9)	580 (5.0)	650 (0.7)	657, 708	7
**SW**	416 (250.6)	514 (13.9)	550 (3.7)	581 (5.9)	650 (0.5)	656, 707	6

B	Absorbance properties λ_{max} (nm) (ε x 10^3 $M^{-1}cm^{-1}$)					Fluorescence propeties	
	Soret	Q_y(1-0)	Q_y(0-0)	Q_x(1-0)	Q_x (0-0)	λ_{max} (nm)	Stokes shift
**DEMI**	418 (255.3)	516 (14.9)	553 (3.7)	581 (5.6)	648 (0.9)	655, 704	7
**HW**	418 (238.7)	517 (12.7)	548 (4.7)	581 (5.2)	650 (0.8)	656, 705	6
**SW**	418 (250.4)	516 (13.6)	553 (4.1)	581 (5.4)	650 (0.7)	656, 705	1

C	Absorbance properties λ_{max} (nm) (ε x 10^3 $M^{-1}cm^{-1}$)					Fluorescence propeties	
	Soret	Q_y(1-0)	Q_y(0-0)	Q_x(1-0)	Q_x (0-0)	λ_{max} (nm)	Stokes shift
**DEMI**	420 (255.0)	517 (14.7)	552 (4.0)	584 (5.5)	650 (0.8)	649, 708	/
**HW**	420 (159.2)	519 (12.6)	553 (5.2)	588 (4.9)	649 (1.4)	649, 708	/
**SW**	420 (177.4)	518 (12.5)	555 (4.5)	587 (4.0)	646 (1.4)	649, 708	3

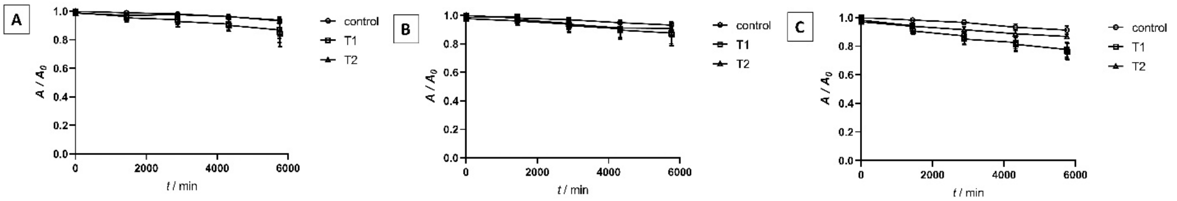

Figure S1. Photostability of TMPyP3 in DEMI (A), SW (B) and HW (C) measured by the decrease of Soret band absorption intensity in a 5-day experiment. T1 represents a 10 μM PS solution in waters of different hardness irradiated for 10 minutes every 24 h over 5 days (411 nm; total light dose 33 J/cm^2), while T2 represents samples irradiated for 10 minutes only on the first day of the experiment, and the stability was measured every 24h for 5 days (411 nm; total light dose 6.6 J/cm^2). Control represents a 10 μM solution kept in the dark for 5 days, while absorbance intensity was measured every 24 h. Data represents average of triplicate measurement with error bars presenting standard deviation.

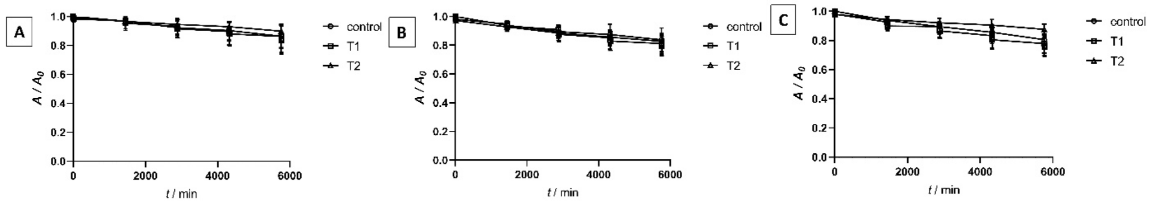

Figure S2. Photostability of TMPyP3-CH_3 in DEMI (A), SW (B) and HW (C) measured by the decrease of Soret band absorption intensity in a 5-day experiment. T1 represents a 10 μM PS solution in waters of different hardness irradiated for 10 minutes with violet light every 24 h over 5 days (411 nm; total light dose 33 J/cm^2), while T2 represents samples irradiated for 10 minutes only on the first day of the experiment, and the stability was measured every 24 h for 5 days (411 nm; total light dose 6.6 J/cm^2). Control represents a 10 μM solution kept in the dark for 5 days, while absorbance intensity was measured every 24 h. Data represents average ± SD.

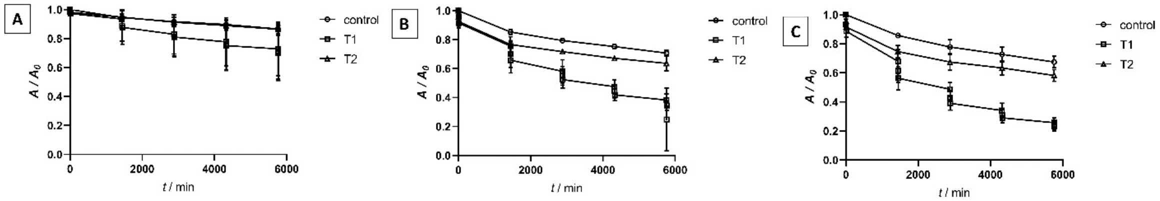

Figure S3. Photostability of TMPyP3-$C_{17}H_{35}$ in DEMI (A), SW (B) and HW (C) measured by the decrease of Soret band absorption intensity in a 5-day experiment. T1 represents a 10 μM PS solution in waters of different hardness irradiated for 10 minutes with violet light every 24 h over 5 days (411 nm; total light dose 33 J/cm^2), while T2 represents samples irradiated for 10 minutes only on the first day of the experiment, and the stability was measured every 24 h for 5 days (411 nm; total light dose 6.6 J/cm^2). Control represents a 10 μM solution kept in the dark for 5 days, while absorbance intensity was measured every 24 h. Data represents average ± SD.

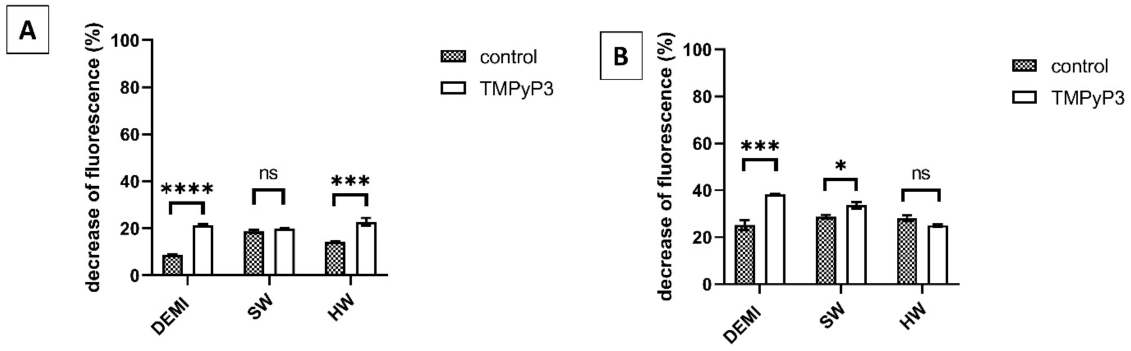

Figure S4. Singlet oxygen production of TMPyP3 (1 μM) measured by photodegradation of ABMDMA (6 μM). For irradiation, a LED-based source of violet light (411 nm) was used with a fluence rate 7 mW/cm^2 (light dose 4.2 J/cm^2) (A) and 11 mW/cm^2 (light dose 6.6 J/cm^2) (B). Control represents photodegradation of ABMDMA (6 μM) without PS. Data is presented as an average of duplicate measurement ± SD; **** $p < 0.0001$, *** $0.0001 < p < 0.001$, * $0.01 < p < 0.05$.

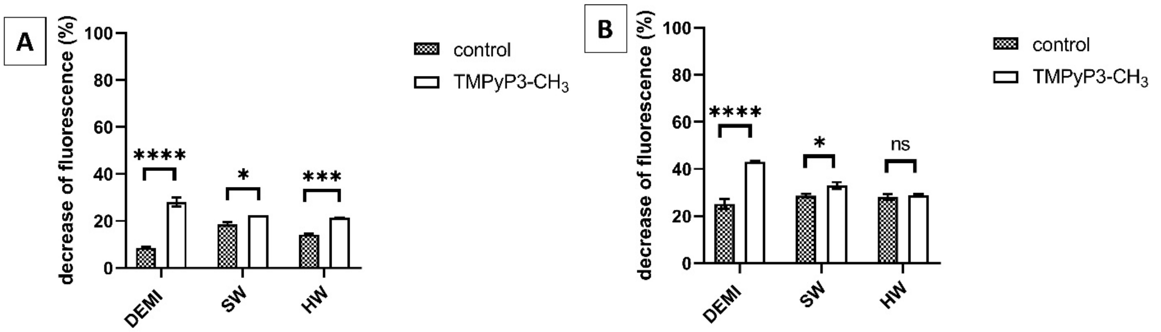

Figure S5. Singlet oxygen production of TMPyP3-CH_3 (1 μM) measured by photodegradation of ABMDMA (6 μM). For irradiation, a LED-based source of violet light (411 nm) was used with a fluence rate 7 mW/cm^2 (light dose 4.2 J/cm^2) (A) and 11 mW/cm^2 (light dose 6.6 J/cm^2) (B). Control represents photodegradation of ABMDMA (6 μM) without PS. Data is presented as an average of duplicate measurement ± SD; **** $p < 0.0001$, *** $0.0001 < p < 0.001$, * $0.01 < p < 0.05$.

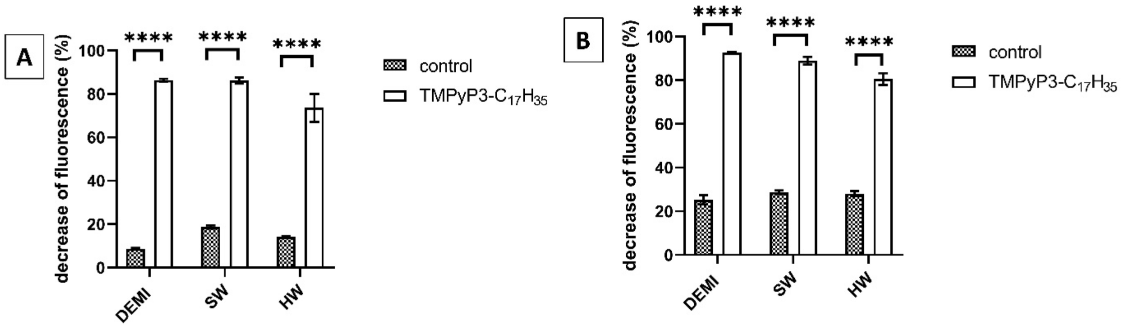

Figure S6. Singlet oxygen production of TMPyP3-$C_{17}H_{35}$ (1 μM) measured by photodegradation of ABMDMA (6 μM). For irradiation, a LED-based source of violet light (411 nm) was used with a fluence rate 7 mW/cm^2 (light dose 4.2 J/cm^2) (A) and 11 mW/cm^2 (light dose 6.6 J/cm^2) (B). Control represents photodegradation of ABMDMA (6 μM) without PS. Data is presented as an average of duplicate measurement ± SD; **** $p < 0.0001$.

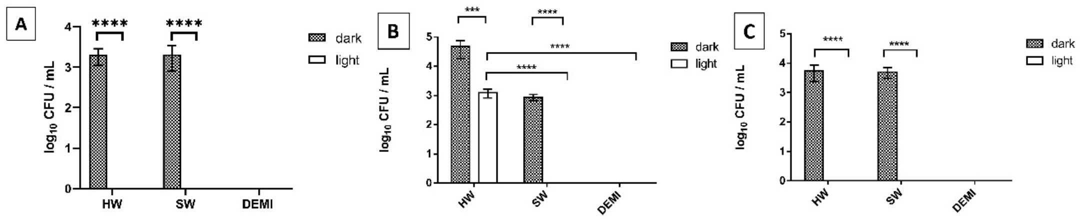

Figure S7. Impact of the PDI on the adhesion of *L. pneumophila* on polystyrene using PSs TMPyP3 (A), TMPyP3-CH_3 (B) and TMPyP3-$C_{17}H_{35}$ (C) in concentration 0.5x MEC in waters of different hardness (DEMI, SW and HW). For irradiation, a LED-based source of violet light was used for 10 min (395 nm, 20 mW/cm^2, 12 J/cm^2). Dark control represents the samples tested with PSs without light irradiation. Data is shown as an average ± SD; .**** $p < 0.0001$, *** $0.0001 < p < 0.001$.

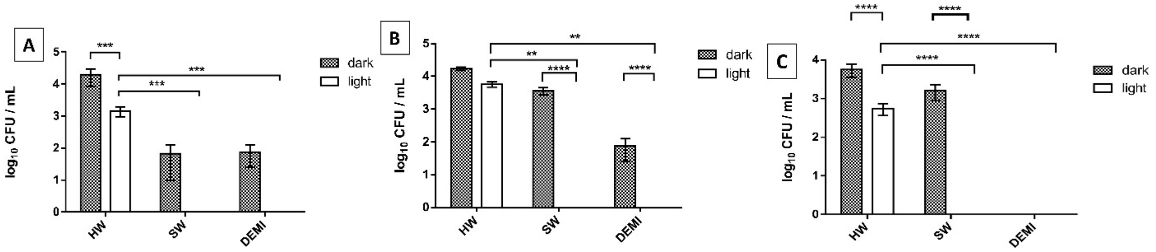

Figure S8. Impact of the PDI on the *L. pneumophila* biofilm formation using PSs TMPyP3 (A), TMPyP3-CH_3 (B) and TMPyP3-$C_{17}H_{35}$ (C) in concentration 0.5x MEC in waters of different hardness (DEMI, SW and HW). For irradiation, a LED-based source of violet light was used for 10 min (395 nm, 20 mW/cm^2, 12 J/cm^2). Dark control represents the samples tested with PSs without light irradiation. Data is shown as an average ± SD; .**** $p < 0.0001$, *** $0.0001 < p < 0.001$, ** $0.001 < p < 0.01$.

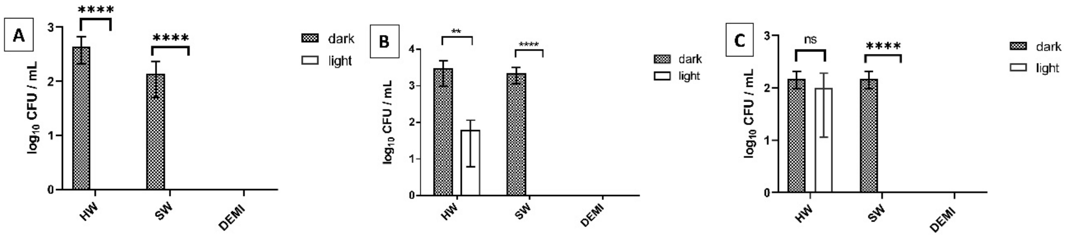

Figure S9. Impact of the PDI on the *L. pneumophila* biofilm destruction using PSs TMPyP3 (A), TMPyP3-CH_3 (B) and TMPyP3-$C_{17}H_{35}$ (C) in concentration 2x MEC in waters of different hardness (DEMI, SW and HW). For irradiation, a LED-based source of violet light was used for 10 min (395 nm, 20 mW/cm^2, 12 J/cm^2). Dark control represents the samples tested with PSs without light irradiation. Data is shown as an average ± SD; .**** $p < 0.0001$, ** $0.001 < p < 0.01$.
